# Supplementary material for: Central versus Peripheral Postcardiotomy Veno-Arterial Extracorporeal Membrane Oxygenation: Systematic Review and Individual Patient Data Meta-Analysis
Source: J Clin Med. 2022 Dec 14;11(24):7406. doi: 10.3390/jcm11247406 (PMC9785985; doi:10.3390/jcm11247406)
Supplement: Supplementary file 1 [file jcm-11-07406-s001.zip › jcm-2064443-supplementary.pdf]

Journal of
Clinical Medicine

MDPI

Central versus Peripheral Postcardiotomy Veno-Arterial Extracorporeal Membrane Oxygenation: Systematic Review and Individual Patient Data Meta-analysis

Supplementary Table S1. PRISMA 2020 checklist.

Section and Topic	Item #	Checklist item	Location where item is reported
		**TITLE**	
Title	1	Identify the report as a systematic review.	1
		**ABSTRACT**	
Abstract	2	See the PRISMA 2020 for Abstracts checklist.	2
		**INTRODUCTION**	
Rationale	3	Describe the rationale for the review in the context of existing knowledge.	3
Objectives	4	Provide an explicit statement of the objective(s) or question(s) the review addresses.	3
		**METHODS**	
Eligibility criteria	5	Specify the inclusion and exclusion criteria for the review and how studies were grouped for the syntheses.	4
Information sources	6	Specify all databases, registers, websites, organisations, reference lists and other sources searched or consulted to identify studies. Specify the date when each source was last searched or consulted.	5
Search strategy	7	Present the full search strategies for all databases, registers and websites, including any filters and limits used.	5
Selection process	8	Specify the methods used to decide whether a study met the inclusion criteria of the review, including how many reviewers screened each record and each report retrieved, whether they worked independently, and if applicable, details of automation tools used in the process.	4,5
Data collection process	9	Specify the methods used to collect data from reports, including how many reviewers collected data from each report, whether they worked independently, any processes for obtaining or confirming data from study investigators, and if applicable, details of automation tools used in the process.	5
Data items	10a	List and define all outcomes for which data were sought. Specify whether all results that were compatible with each outcome domain in each study were sought (e.g. for all measures, time points, analyses), and if not, the methods used to decide which results to collect.	5
	10b	List and define all other variables for which data were sought (e.g. participant and intervention characteristics, funding sources). Describe any assumptions made about any missing or unclear information.	5
Study risk of bias assessment	11	Specify the methods used to assess risk of bias in the included studies, including details of the tool(s) used, how many reviewers assessed each study and whether they worked independently, and if applicable, details of automation tools used in the process.	5
Effect measures	12	Specify for each outcome the effect measure(s) (e.g. risk ratio, mean difference) used in the synthesis or presentation of results.	6

Section and Topic	Item #	Checklist item	Location where item is reported
Synthesis methods	13a	Describe the processes used to decide which studies were eligible for each synthesis (e.g. tabulating the study intervention characteristics and comparing against the planned groups for each synthesis (item #5)).	5,9
	13b	Describe any methods required to prepare the data for presentation or synthesis, such as handling of missing summary statistics, or data conversions.	6
	13c	Describe any methods used to tabulate or visually display results of individual studies and syntheses.	9
	13d	Describe any methods used to synthesize results and provide a rationale for the choice(s). If meta-analysis was performed, describe the model(s), method(s) to identify the presence and extent of statistical heterogeneity, and software package(s) used.	6
	13e	Describe any methods used to explore possible causes of heterogeneity among study results (e.g. subgroup analysis, meta-regression).	6
	13f	Describe any sensitivity analyses conducted to assess robustness of the synthesized results.	6
Reporting bias assessment	14	Describe any methods used to assess risk of bias due to missing results in a synthesis (arising from reporting biases).	5
Certainty assessment	15	Describe any methods used to assess certainty (or confidence) in the body of evidence for an outcome.	-
**RESULTS**			
Study selection	16a	Describe the results of the search and selection process, from the number of records identified in the search to the number of studies included in the review, ideally using a flow diagram.	Figure 1
	16b	Cite studies that might appear to meet the inclusion criteria, but which were excluded, and explain why they were excluded.	Supplementary table 2
Study characteristics	17	Cite each included study and present its characteristics.	9
Risk of bias in studies	18	Present assessments of risk of bias for each included study.	9
Results of individual studies	19	For all outcomes, present, for each study: (a) summary statistics for each group (where appropriate) and (b) an effect estimate and its precision (e.g. confidence/credible interval), ideally using structured tables or plots.	Figure 2
Results of syntheses	20a	For each synthesis, briefly summarise the characteristics and risk of bias among contributing studies.	9
	20b	Present results of all statistical syntheses conducted. If meta-analysis was done, present for each the summary estimate and its precision (e.g. confidence/credible interval) and measures of statistical heterogeneity. If comparing groups, describe the direction of the effect.	13
	20c	Present results of all investigations of possible causes of heterogeneity among study results.	9
	20d	Present results of all sensitivity analyses conducted to assess the robustness of the synthesized results.	7-10
Reporting biases	21	Present assessments of risk of bias due to missing results (arising from reporting biases) for each synthesis assessed.	-
Certainty of evidence	22	Present assessments of certainty (or confidence) in the body of evidence for each outcome assessed.	7-10
**DISCUSSION**			

Section and Topic	Item #	Checklist item	Location where item is reported
Discussion	23a	Provide a general interpretation of the results in the context of other evidence.	13-16
	23b	Discuss any limitations of the evidence included in the review.	13-16
	23c	Discuss any limitations of the review processes used.	13-16
	23d	Discuss implications of the results for practice, policy, and future research.	13-16
**OTHER INFORMATION**			
Registration and protocol	24a	Provide registration information for the review, including register name and registration number, or state that the review was not registered.	3
	24b	Indicate where the review protocol can be accessed, or state that a protocol was not prepared.	3
	24c	Describe and explain any amendments to information provided at registration or in the protocol.	3
Support	25	Describe sources of financial or non-financial support for the review, and the role of the funders or sponsors in the review.	21
Competing interests	26	Declare any competing interests of review authors.	21
Availability of data, code and other materials	27	Report which of the following are publicly available and where they can be found: template data collection forms; data extracted from included studies; data used for all analyses; analytic code; any other materials used in the review.	21

Supplementary Table S2. Studies potentially suitable for this analysis, whose data was not provided by the authors.

No.	Excluded studies
1	Risk factors of acute renal injury and in-hospital mortality in adult patients with postcardiotomy cardiogenic shock requiring veno-arterial extracorporeal membrane oxygenation: utility of MELD-XI score. Perfusion. 2022 Jul;37(5):505-514.
2	Risk factors for agitation and hyperactive delirium in adult postcardiotomy patients with extracorporeal membrane oxygenation support: an observational study. Perfusion. 2020 Sep;35(6):534-542.
3	The Effect of Simultaneous Renal Replacement Therapy on Extracorporeal Membrane Oxygenation Support for Postcardiotomy Patients with Cardiogenic Shock: A Pilot Randomized Controlled Trial. J Cardiothorac Vasc Anesth. 2019 Nov;33(11):3063-3072.
4	The early dynamic behavior of lactate is linked to mortality in postcardiotomy patients with extracorporeal membrane oxygenation support: A retrospective observational study. J Thorac Cardiovasc Surg. 2015 May;149(5):1445-50.
5	A Simple Scoring System to Predict Survival after Venoarterial Extracorporeal Membrane Oxygenation. J Extra Corpor Technol. 2019 Sep;51(3):133-139.
6	Extracorporeal membrane oxygenation for refractory cardiogenic shock: patient survival and health-related quality of life. Eur J Cardiothorac Surg. 2019 Apr 1;55(4):780-787.
7	Benefits of Intra-Aortic Balloon Pump Support in Patients with Postcardiotomy Shock Requiring Venoarterial Extracorporeal Membrane Oxygenation. Life (Basel). 2022 Aug 5;12(8):1195.
8	Outcomes after extracorporeal life support for postcardiotomy cardiogenic shock. J Card Surg. 2019 Feb;34(2):74-81.
9	The Early Introduction of Extracorporeal Membrane Oxygenation for Postcardiotomy Cardiogenic Shock Does Not Improve 30-Day Mortality Rates in Low-Volume Centers. Cureus. 2022 Feb 21;14(2):e22474.

10	Venoarterial extracorporeal membrane oxygenation for postcardiotomy cardiogenic shock-A six-year service evaluation. Artif Organs. 2020 Jul;44(7):709-716.
11	Experience with Extracorporeal Life Support for Cardiogenic Shock in the Older Population more than 70 Years of Age. ASAIO J. 2017 May/Jun;63(3):279-284.
12	A Standardized Approach Improves Outcomes of Extracorporeal Membrane Oxygenation for Postcardiotomy Shock. ASAIO J. 2021 Oct 1;67(10):1119-1124.
13	Early venoarterial extracorporeal membrane oxygenation improves outcomes in post-cardiotomy shock. J Artif Organs. 2021 Mar;24(1):7-14.
14	Clinical outcomes of venoarterial extracorporeal life support in 462 patients: Single-center experience. Artif Organs. 2020 Jun;44(6):620-627.
15	Contemporary management of postcardiotomy cardiogenic shock: results of a specialized care team. Rev Esp Cardiol (Engl Ed). 2021 Mar;74(3):275-278.
16	Clinical Features and outcomes in adults with cardiogenic shock supported by extracorporeal membrane oxygenation. Am J Cardiol. 2015 Nov 15;116(10):1624-30.
17	Concomitant ECMO And IABP Support in Postcardiotomy Cardiogenic Shock Patients. Heart Lung Circ. 2021 Oct;30(10):1533-1539.
18	A comparison of existing risk prediction models in patients undergoing venoarterial extracorporeal membrane oxygenation. Heart Lung. 2020 Sep-Oct;49(5):599-604.
19	Survival following venoarterial extracorporeal membrane oxygenation in postcardiotomy cardiogenic shock adults. Perfusion. 2020 Nov;35(8):747-755.
20	Prognostic impact of blood product transfusion in VA and VV ECMO. Perfusion. 2019 Apr;34(3):246-253.
21	Risk Factors for Developing Severe Acute Kidney Injury in Adult Patients With Refractory Postcardiotomy Cardiogenic Shock Receiving Venoarterial Extracorporeal Membrane Oxygenation. Crit Care Med. 2020 Aug;48(8):e715-e721.
